# Conceptualising Rugby League Performance Within an Ecological Dynamics Framework: Providing Direction for Player Preparation and Development

**DOI:** 10.1186/s40798-021-00375-x

**Published:** 2021-11-24

**Authors:** Tannath J. Scott, Colin E. Sanctuary, Matthew S. Tredrea, Adrian J. Gray

**Affiliations:** 1 Performance Department, New South Wales Rugby League, Sydney, Australia; 2 Carnegie Applied Rugby Research (CARR) Centre, Carnegie School of Sport, Leeds Beckett University, Leeds, UK; 3 School of Science and Technology, University of New England, Armidale, NSW Australia; 4 School of Education, University of Newcastle, Newcastle, Australia; 5 Discipline of Sport and Exercise Science, School of Allied Health, Human Services and Sport, La Trobe University, Melbourne, Australia; 6 School of Health and Behavioural Sciences, University of Sunshine Coast, Sunshine Coast, Australia; 7 College of Engineering, Swansea University, Swansea, UK

**Keywords:** Performance analysis, Team sports, LTAD, Learning design, Strength and conditioning

## Abstract

Across team sports, it is critically important to appropriately define, evaluate and then aptly describe individual and team performance. This is of particular significance when we consider that performance models govern the direction of player preparation (short term) and development (long term) frameworks. Within the context of rugby league, this has traditionally been undertaken through hierarchical and linear processes. Such approaches have resulted in research and performance analysis techniques which aim to support these operational outcomes. Yet, these methods may deliver limited application on *how* or *why* match-play unfolds and therefore might be sub-optimal in providing insights to truly support coaches. In this paper, we propose the conceptualisation of rugby league performance through the lens of ecological dynamics, which may offer a different view to this traditional approach. We propose that this approach eliminates the silos of disciplinary information (e.g. technical, physical and medical) that may currently exist, allowing for a holistic approach to performance, preparation and development. Specifically, we consider that through the implementation of this ecological approach, all performance coaches (technical, physical and medical) may (co-)design learning environments that more collaboratively develop players for rugby league match-play. As a result, we put forward a new rugby league performance model from which preparation and development programs can be anchored toward. We conclude the paper by offering practical examples where these concepts are contextualised within the landscape familiar to practitioners working within rugby league.

## Key Points

- Current player preparation and development frameworks are anchored toward successful team performance, primarily implementing hierarchical performance models in an operational approach.
- Through the lens of ecological dynamics, we propose a re-conceptualisation of this approach; providing an updated performance model for rugby league (and team sports), supporting direction for preparation and development frameworks.
- In this framework, we propose a change in how we view coaching (across domains; e.g., technical, physical), shifting from a solution provider, to one of a learning environment designer that places the athlete-environment interaction at its core.
- Two examples are presented to provide practitioners with the contextual information to allow for the appropriate implementation of the proposed model in rugby league.

## Introduction

“If you change the way you look at things, the things you look at change” – Wayne W. Dyer

At the elite level, rugby league players are prepared by multidisciplinary teams to meet the demands of match-play, which are multi-faceted and varied between positional groups [1]. Positional demands are often described in terms of the tactical, technical and physical/physiological requirements of play [2]. Accordingly, these are often found as key themes of work in player preparation (short term) and development (long term) programmes, alongside perceptual, mental/psychological skills, injury prevention/management and player well-being [2]. As central components of high-performance systems, it is unsurprising that several reviews have collated works on the physical characteristics, physiological capacities, technical and tactical abilities, match-demands, strength and conditioning practices, as well as injury epidemiology and aetiology in rugby league [1–10]. Collectively, these works have guided coaching and performance practices.

Successful team performance lies at the core of rugby league player preparation and development, with programmes governed by the performance models (and supportive research outcomes) employed. Yet a definitive model of rugby league performance does not exist. Instead, it can only be assumed that to date, sports practitioners have combined and assimilated rugby league literature with their own anecdotal experience, to form an understanding of rugby league performance to inform practice. As previous reviews in rugby league are typically isolated summaries of descriptive studies, it would appear that practitioners' current understanding of rugby league performance (i.e. traditional approach) contains limited considerations of the performance interactions across disciplines [11]. To the authors' knowledge, no literature has aimed to summate current evidence related to rugby league performance (through the lens of this traditional approach), while assessing the potential impact this information may have on individual and/or team performance. Indeed, to better understand the varied and complex ways in which rugby league players combine as a team and interact to play the game, clear definitions of measurable constructs synonymous with ‘rugby league performance’ are required. How each of these constructs combine to explain performance outcomes (success/failure) appears paramount to the appropriate design of player preparation and development programmes. Yet, whether current methods used to identify, evaluate and *describe* performance, preparation and development models in rugby league are appropriate, requires further investigation.

## Part 1: A Traditional Approach to Performance, Preparation and Development

### Understanding Rugby League Performance: The Operational Model

Attributable to competition structures and rules of play, complex invasion sports are considered to abide by a traditional hierarchical structure [12, 13], whereby lower-order events (e.g. effective individual skill execution) are required to be performed for higher-order events to occur (e.g. scoring points, winning). In rugby league, winning a premiership/competition is the overarching objective and sits atop the performance hierarchy. The competition structure then logically dictates that to win the premiership, a team must win more matches than others. Within the hierarchy, the rules of play logically dictate the only way a team can win games is to accumulate more points than their opponent. Hence, the irrefutable fact is that, in semi- and professional rugby league: ladder position, match outcome (win/loss/draw), point differential (scored/conceded), and possession and field position (intertwined) stand as objective measures of rugby league performance.

**Fig. 1:**
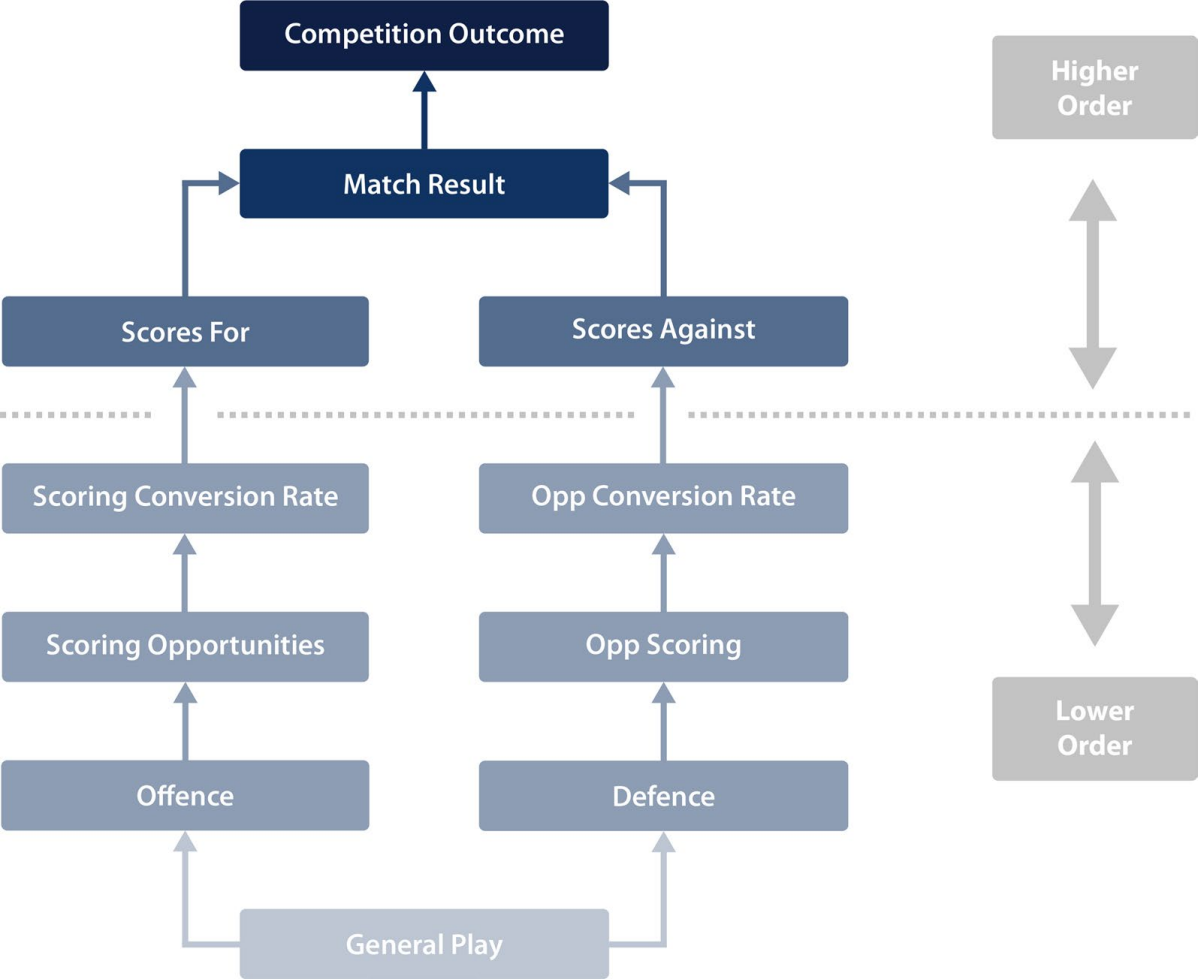
A hierarchical performance model for invasion team sports (adapted from Gerrard [12], with permission). Opp: Opposition

Figure 1 provides the basis by which we currently conceptualise performance in rugby league. Performance analysis and research methods have traditionally aimed to support and refine this performance model through operational appraisal (e.g. notational analysis) and reporting of match-play outcomes (e.g. locomotor analysis, match statistical analysis) [14, 15]. As preparation (short term) and development (long term) programmes are developed around supporting individual and team performance, these frameworks are commonly conceptualised using linear processes. Such frameworks are established through a systematic progression of identifying and evaluating performance alongside the relevant training and development required to maximise desired goals (i.e. needs analysis). For example, we may consider *individual capabilities* are developed and optimised to drive *individual actions* [16, 17], which lead to preferable *team actions* and *play outcomes* (within-match) or match/seasonal outcomes (between-match) [14, 15]. However, this conceptualisation of rugby league performance, and therefore preparation and development, is often centred on established disciplines (i.e. technical and tactical, strength and conditioning, medical) to allow for the effective organisation of this linear information [18, 19]. As such, the existing literature has identified *key performance indicators* (KPIs) that exist within these disciplines, to understand rugby league performance and help support athlete preparation and development.

### Rugby League Performance Indicators

As identified earlier, competition/game rules dictate team response variables in the highest level of the hierarchy. In rugby league competitions, it is commonplace for ladder position, match outcome (win/loss/draw) and point differential (scored/conceded) to be used as team-level KPIs [14, 15, 20, 21]. This section aims to identify rugby league specific (within-match) KPIs at the lower levels of the performance hierarchy, i.e. *‘play outcomes’, ‘actions’* and *‘individual capabilities’.* It is assumed that the reader has an understanding of the game of rugby league. If not, detailed descriptions of the rules and history of rugby league can be found elsewhere [22, 23]. While peer-reviewed rugby league research has existed since the late 1970s, an exponential increase in study outputs occurred in the late 1990s and early 2000s. Notably, this corresponds to the time when rugby league became fully professional in Australia and New Zealand (known as the National Rugby League competition, NRL; 1999) and the UK (known as the Super League competition, SL; 1996) [22]. As such, this section will only present the literature and evidence from 2000 onwards.

#### Play Outcomes

Like higher level KPIs, some ‘*play outcome*’ level KPIs may also be inferred from game rules and structure of play. Research within rugby league has demonstrated, across elite [14, 15], sub-elite [24] and junior-elite [25] levels that successful teams gained more metres in attack than their less successful counterparts. Kempton and colleagues [21] used a probabilistic model to estimate the expected point outcome for a given position and situation when in possession. They found it was essential to promote the ball down-field each tackle to preserve or improve the expected point equity. For example, if the team in possession did not cross the midfield by the fourth tackle, the ensuing play had a resultant negative point equity. Put simply, that team is not expected to score any points during that possession. Indeed, it stands to reason that while gaining more territory appears advantageous when in possession, limiting the progression of the opposition is equally effective when defending [24]. It has been shown that having possession of the ball deeper in one’s own defensive zone is associated with diminished point estimation [21]. That is, the likelihood of scoring on a specific play is substantially lower the further you are away from the try-line. Therefore, containing the opposition to their own half, as well as forcing their possession to start close to their own try-line, appears beneficial. This is also reflected in coaching philosophies, with defensive (line speed) and kick pressure spoken of as a key element in defensive strategies [26]. Significantly, recent analysis has supported these long-standing philosophies, demonstrating kick pressure [27] and defensive quickness [28] as influential actions that impact match and season outcomes.

#### Individual and Team Actions

Several studies have aimed to classify and determine the relative importance of individual and team *actions* for rugby league performance. Woods et al. [15] examined the use of team performance indicators to explain match outcome and subsequent ladder position across the 2016 NRL season. Using a conditional interference classification tree, it was revealed that five performance indicators (try assists, all attacking run metres, line breaks, number of dummy half runs and offloads) effectively explained match outcome, correctly classifying 91% of wins and 66% of losses. These authors reported try assists and attacking run metres (> 4 and > 1340 m, respectively) to have the greatest probability of winning (98%), while a combination of try assists (≤ 2), attacking run metres (≤ 1450 m) and line breaks (≤ 4) led to a lower probability of winning (5%). Taken collectively, this study supports past research [14, 21, 24, 27–29] recognising that more efficiently moving the ball down-field and having playmakers able to assist with line breaks and scoring situations is beneficial to performance. Further to this, using principal component analysis (to cluster like performance indicators), Parmar and colleagues [28] indicated that if a team increased ‘amount of possession’ (e.g. number of plays, metres gained) and ‘making quick ground’ (e.g. tackle busts, supported breaks) component scores, they were more likely to win. Notably, this analysis technique separated ‘possession’ and ‘speed of play’ (e.g. line breaks, dummy half running) as two distinct components, signalling that these indicators may act as two separate beneficial functions of performance. Using a similar methodological design, Wedding et al. [27] demonstrated that controlled ball possession and player efforts (e.g. kick pressure and supporting runs) to impacted season outcomes, supporting these findings. Lastly, other, but not all [14], work has highlighted the importance of an effective kicking game with some reporting a positive association between match outcome and match-play kick metres in both elite [14, 15] and sub-elite [24] rugby league.

Various analyses have identified a relationship between defence proficiency, discipline and match outcome. Woods et al. [15] revealed teams with fewer missed tackles, finished higher at the conclusion of the competitive season. This outcome is reinforced by additional research demonstrating that successful NRL teams presented a substantially higher effective tackle percentage and had fewer missed tackles than less successful teams [14, 30]. Poor discipline (measured by the number of errors and penalties) relinquishes both possession and field position, thereby adversely influencing performance. For example, when a team gives up possession of the ball, due to an error, the opposition has been shown to have a greater chance of scoring (~ 15% of occurrences, accounting for 40% of all tries scored), due to the advanced position of the ball [21]. This is a near fourfold increase compared to relinquishing possession through a kick. This may in part explain why Gabbett and Hulin [30] identified that more successful teams completed fewer offloads than less successful teams, electing not to risk the loss of possession. Similarly, when a team in possession is awarded a penalty or can force a drop out, the likelihood of scoring is dramatically improved (19% and 21%, respectively, on the ensuing possession) due to the repeated possession and likely attacking field position [21]. Taken together, teams can minimise opposition scoring through improved tackling effectiveness, as well as a reduction in errors and penalties. Given the dramatic difference in these outcomes, it is evident that successful teams are characterised by sound defence and demonstrating exceptional discipline.

Unsurprisingly, the aforementioned performance indicators vary slightly between age groups (e.g. senior vs junior) and domestic competitions (NRL vs. SL). In a comparative analysis between NRL and National Youth Competition (NYC; replica U20s competition running from 2008 to 2017), it was demonstrated that NRL games were characterised by a greater number of runs, tackles, and a lower amount of tackle breaks than NYC [25]. Similarly, in the UK-based competitions, SL (elite) and academy-based match-play may be differentiated through both physical and technical/tactical performance indicators, while positional differences (backs, forwards) are also apparent across levels of competition [31]. These findings suggest that match-play at lower levels of competition (e.g. junior rugby league) is played with a different tactical emphasis and/or execution. [25]. Equally, differences between the NRL and SL have been reported [20, 29]. In a similar analysis to that reported above [25], seven of eleven KPIs investigated between NRL and SL, showed large to very large effects, demonstrating differences between game-play styles within each competition. These data showed that SL displayed an increase in line breaks, errors, tackles, and dummy-half runs, compared to the NRL [20]. The discrepancies between these competitions may be most associated with an increased amount of missed and ineffective tackles in junior and SL competition, respectively, potentially leading to an increase in tackle breaks and line breaks [20, 25]. Lastly, it appears the relative influence of these performance indicators changes across positions over time [32], demonstrating a constant evolution of game-style; importantly, such outcomes may have impact on how we evaluate and identify individual and team performance (year on year).

#### Individual Capabilities

Through the hierarchical conceptualisation of rugby league performance, individual capabilities (i.e. lower-body strength and power, aerobic power, draw and pass) may provide the foundation which drives our performance outcomes. On this basis, coaches aim to systematically develop these qualities in respect of the player’s strengths/weaknesses, the match-play demands of their position [33], to aid muscle integrity [34], improve body composition [35], among others. Indeed, it is commonly regarded that rugby league players require a broad range of physical, physiological, technical and perceptual qualities [2]. Within the linear process of the hierarchical model, it is important to determine the relative importance of the individual capabilities which relate to the KPIs highlighted above.

##### Physical and Physiological Qualities

The link between physical/physiological qualities and match-play *actions* is still ambiguous, with a disproportionate distribution of current research focussed on ‘defensive’ *actions*. Further, the majority of these data are taken from sub-elite (semi-professional) and junior (both elite and sub-elite) players, providing challenges and limitations when aiming to design conclusive player development frameworks (from junior to elite).

Research linking physical qualities to influential ‘defensive’ *actions* has primarily focussed on tackling ability [16, 17]. Here, evidence demonstrates that tackling ability can distinguish levels of competition [16, 17], establishing some construct validity behind its actual and/or perceived contribution towards ﻿*play outcomes*. One study examining tackling ability of professional rugby league players reported two physical qualities (acceleration; *r* = 0.41 and lower-body power; *r* = 0.38) to significantly (*p* ≤ 0.05) correlate with tackling ability [16]. Further, lower-body power, along with measures of acceleration and speed, has been significantly associated with tackles completed (positive) and the proportion of missed tackles (negative) [36]. Studies examining the tackling ability of semi-professional and junior rugby league players have reported similar findings, showing strong relationships with tackling proficiency and lower-body explosiveness. Indeed, the majority of these studies, though not all [37], report the influence of increased acceleration [16, 36, 38, 39], lower-body strength [40, 41] and power [16, 36, 39], speed [36], agility [38], and to a lesser extent, upper-body strength and power [40], on the ability to tackle effectively. For example, improvements in maximal and relative lower-body strength have been demonstrated to significantly improve tackling ability [42] and dominant (winning) tackles performed [41]. Further, Gabbett [43] reported that lower-body strength was found to be the only physical quality correlated to tackling ability, under fatigue, in semi-professional rugby league. Lastly, it appears that improved anthropometrical qualities (lower skinfold, and improved lean muscle mass) also assist in improved tackling ability [16, 38]. Collectively, these findings provide clear evidence for an interaction between physical qualities and defensive skill execution.

In contrast to these studies, a small body of research exists examining interactions between physical qualities and the execution of ‘offensive’ *actions*. From this limited research, it appears that explosive qualities are of significance when in attack. Gabbett et al. [36] reported speed (40-m time) to have a moderate (*r* = − 0.42) association with tries scored. Similarly, in elite junior rugby league players, acceleration, in accordance with gains in body mass, substantially influenced the ability to successfully carry to the ball into the defensive line (and ‘win’ the play the ball) [37].

##### Technical and Perceptual Qualities

Technical and perceptual qualities are commonly cited as important capabilities for successful rugby league performance [2, 31, 36]. With specific regard to the offensive and defensive aspects of un/successful performance [15, 24, 30] the reasons behind these occurrences and the respective in/appropriate decision-making may be important to understand. Technical (and physical) qualities have been highlighted as distinguishing characteristics in both the selection and effective playing performance of elite rugby league players [36, 44]. This is supported by Kempton et al. [45] who reported that skill ratings (rated 1–5 by expert coaches) and involvements were higher in the first 5 min of each rugby league game, suggesting an interplay between these qualities. Although this work is retrospective and match demands have been shown to evolve [30], the work reinforces the importance of the skilled execution of an *action* in relation to performance. Gabbett and Ryan [17] highlight this association between performance and the application of skill through the assessment of in/appropriate decision-making. They demonstrated that a one-on-one criteria-based tackling drill (assessing tackling technique) could discriminate between professional and semi-professional rugby league players. Furthermore, higher-skilled tacklers made a greater proportion of dominant tackles and missed a smaller proportion of tackles compared to lesser-skilled tacklers [17].

When assessing specific rugby league skills (e.g. tackling, draw and pass), current research suggests that perception and decision-making moderate performance; highlighting an expert’s advantage appears to be perceptual [46]. Indeed, improved perceptual skills (e.g. dual task performance) [36, 47] have been suggested to assist elite rugby league players during the increasingly important (e.g. draw and pass) and complex tasks that occur in match-play, which are known to require greater attentional demands [48]. This may also be aided by improved pattern recall and prediction ability, which has been shown to be associated with line break assists in elite players [36]. Indeed, elite rugby league players have been shown to focus on different movements and cues during rugby league movement patterns when compared to novices [49]. It has also been reported that the highest error rates occurred when defending the goal line, where the opposition is likely to be more expansive in their play [50]. This research indicates that the ability to tackle effectively is vital for rugby league performance, while suggesting a player’s perception and decision-making are also key. Yet to date, much of this *technical and perceptual qualities-*based research has been primarily undertaken under constructs founded on the theoretical framework of information processing [46], which may support this traditional approach. Given preparation and development programmes likely encompass nonlinear processes, there may also be value in reconceptualising these outcomes, utilising a framework that allows for insights on the important (complex systems) behaviours that may emerge between individuals, the environment and the task.

### Conclusion and Limitations to an Operational Approach

It is apparent that by utilising current approaches to identify rugby league performance we are able to operationally describe team and individual actions that influence match-play outcomes. In addition, we may establish individual capabilities that have association with the selection of identified influential actions and outcomes. Due to the evolution of match-play [32], these actions and capabilities may differ year to year; however, there appear to be a few that are associated more regularly in the literature (Table 1). As such, using this approach, coaches and performance staff may aim to focus on these in preparation and development planning/programming [20, 31].

**Table 1:** Individual and team actions and individual capabilities shown to relate positively to preferential outcomes in rugby league match-play

		Offence	Defence
Individual and team actions		+ + + Tackle Breaks [14, 15, 27, 28, 30] + + Line Breaks [15, 27, 28] + + Kick Metres [14, 15, 24] + Supporting Runs [27, 28] + Dummy Half Runs [15] − − − Errors [14, 15, 21, 27, 28] − Offloads [15, 30]	− − − Missed Tackles [14, 15, 30] + + + Tackle Effectiveness [14, 27, 30] + Pressure (Line Speed) [26, 28] + Kick Pressure [26, 27]
Individual capabilities	Physical/physiological	+ Speed [36] + Acceleration [37] + LB Power [37]	+ + + Acceleration [16, 36, 38, 39] + + + LB strength [40–43] + + LB power [16, 36, 39] + Agility [38] + Speed [36]
	Technical/perceptual	+ + Dual Task Performance [36, 44, 47, 48] + + Draw and Pass Proficiency [36, 44, 48] + Pattern Recall and Prediction Ability [36]	+ + + Tackling Ability [17, 44]

+: greater, increased, improved; −: lesser, decreased, lower; LB: lower body

However, while statistical modelling can be used to determine relationships between specific aspects of play and higher-order performance indicators, this approach naturally biases higher-order events [12], an outcome known as *the weighting problem*. Though this can be (somewhat) accounted for, without careful analysis, it can lead to futile findings (e.g. try assists have a strong relationship with scoring tries) which are at odds with coaching philosophy, i.e. where players are more frequently coached to execute patterns of play functioning to achieve a specific goal (e.g. gain field position). As a result, notational analysis (or similar forms of performance analysis) that feeds hierarchical models from the bottom up have been criticised for their limited ability to predict performance and/or explain behaviour [51]. This is demonstrated in Table 1, which offers limited insights into *how?* and *why?* individual capabilities determine play outcomes. The associations between *individual capabilities* and KPIs are also less apparent when examining offensive actions. This may be due to the multidimensional nature of offense, whereby players are able to largely dictate the state of play (e.g. ball movement, creativity) as opposed to the mostly reactive nature of defence (where limited degrees of freedom are available). As a result, it is likely that when in attack, players can work together to maximise the expression of their individual capabilities in a variety of beneficial ways that are not currently captured by routine testing. Collectively these outcomes demonstrate that viewing rugby league performance as a simple cause (individual capabilities) and effect (play outcomes) relationship is insufficient. This outcome may in part explain the non-descriptive culmination of Gerrard’s performance model (‘general play’; Fig. 1). Indeed, while some ‘coherent frameworks’ have been proposed [52–54] (based on the exploration of spatio-temporal data), these are still largely operational by nature, limiting their application in understanding team behaviour (the *how* and *why* of Gerrard’s *general play*) [55].

Another limitation with the operational approach of analysis is that it provides information without specific identification of, and reference to the constraints that may shape actions and outcomes. For example, while coaching staff aim to develop individual capabilities to improve positive actions/outcomes, other constraints also naturally change over time (i.e. the *task* and *environment*). Indeed, changes in the task may be evident through alterations in match-play rules (e.g. change in interchange numbers, inclusion of shot-clock), some of which have been shown to impact physical activity cycles and evolution of playing styles [29, 30, 56]. The evolution of match-play (i.e. task) may also be influenced by the successful adaptation and execution of tactical planning. Woods and colleagues [56] were able to characterise differences (or specifically, dissimilarities) in playing styles (albeit through KPIs) in the NRL from 2005 to 2016. These data revealed that teams played with a similar ‘style’ between 2005 and 2011. Yet from the 2012 to 2016 seasons there was a substantial shift in the way teams played, with teams adopting a more free-flowing style of rugby league [56], a notion supported by others [30]. This is punctuated by the NRL finalists who displayed the most dissimilar profiles, indicating that the most successful teams could evolve at a faster rate, with a ‘follow the leader’ pursuit evolving from others [27, 56] (also witnessed in SL [20] and junior elite [25] competitions). Similar findings are also evident with changes to the environment (e.g. weather, location) with differences in playing styles and tactics apparent between the NRL and SL [29]. It is evident that changes to the *task* and *environment* have a significant impact on the operational performance, preparation and development frameworks to be employed.

Taken together, the traditional approach (and associated operational models) used to define and evaluate rugby league performance may be limited due to the restricted explanation of behavioural emergence. This may be of importance to practitioners who are in search of a conceptual model that aims to more appropriately encapsulate the complexity of these adaptive systems. Indeed, while commonly used operational models may successfully diagnose performance outcomes (to varying degrees), they may diminish richer insights into the interaction between the *individual* and the *environment*, thereby often failing to recognise the ‘human’ element of performance. This may be pertinent when creating preparation and development programs, as a greater understanding of *how* and *why* individual capabilities (what we develop) are un/successfully expressed on the field may better guide the prescription of this framework. As such, by disregarding the interaction between the *individual*, the *task* and the *environment*, we may constrain the outcomes of our performance model, limiting the appropriate development and preparation of players. Evidently, to appraise the varied and complex ways in which rugby league players combine as a team and interact during general play, an alternative, robust theoretical framework is needed. Indeed, it seems imperative that ‘general play’ (or team behaviour) is acknowledged as a complex neurobiological system composed of many interacting parts [55, 57] and evaluated accordingly, through explanation of team behaviour on a macroscopic level (e.g. team coordination) prior to identifying the contributions of relevant dynamical components (e.g. individual player movements) [55].

Currently, there are a number of theoretical models which may be conceptualised to describe team behaviours. These theoretical perspectives typically include socio-cognitive (e.g. shared knowledge theory), enactive (e.g. participatory sense-making hypothesis) and dynamic systems approaches (e.g. ecological dynamics theory) [55, 58]. Currently, no approach has been unanimously accepted, though the implementation of such a framework to evaluate team behaviours in rugby league appears useful. A limitation of the socio-cognitive approach in team sports is that it is grounded on rational models of decision-making [58], whereby precise outcomes require immense computational power and are driven by linear processing, disregarding the dynamic constraints experienced by performers [58, 59]. In contrast, a dynamical systems approach to team sport performance aims to describe how coordinated movement patterns develop, persist and adapt [55]. When combined with ecological psychology theory we may garner a greater insight into the mathematical systems that describe behaviour [60]. Indeed, it is suggested that ecological dynamics explanations of team behaviours can surpass operational ‘performance confirmation’, overcoming the aforementioned limitations [55]. As a result, this approach represents a conceptual framework that may allow for an appropriate blend of analytical and behavioural insights, offering a deeper evaluation of rugby league performance, warranting further exploration.

## Part 2: An Ecological Dynamics Approach to Performance, Preparation and Development

“I adapted an antiquated style and modernized it to something that was efficient… I never imagined it would revolutionize the event” – Dick Fosbury

### Ecological Dynamics and Team Sports Performance

**Fig. 2:**
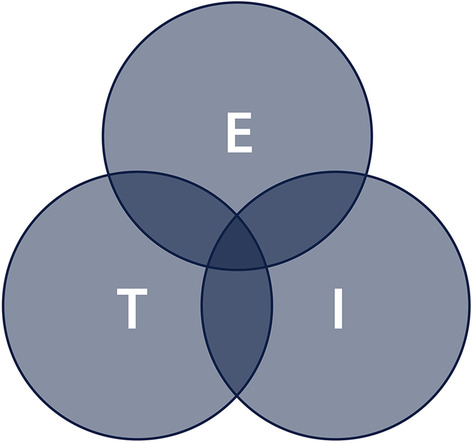
A Venn diagram depicting the interplay between (E) the environment, (T) the task being performed; and (I) the individual organism (player)

**Fig. 3:**
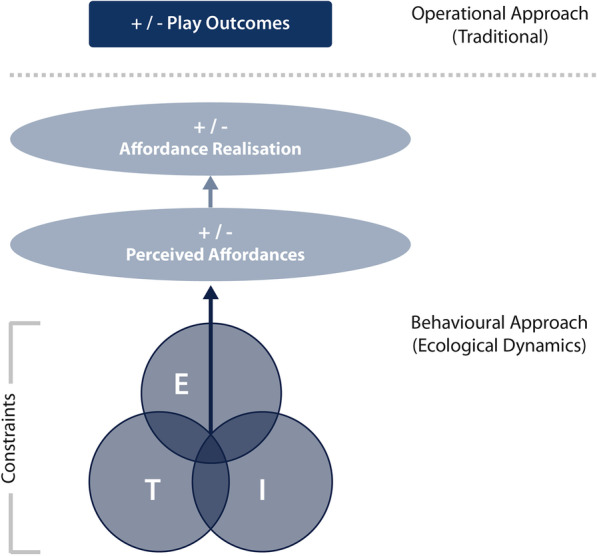
Theoretical basis of an individual player’s behavioural regulation within an ecological dynamics framework. (E) The environment; (T) the task being performed; and (I) the individual player

Ecological dynamics is a theory of motor behaviour that has previously been applied to human movement and the analysis of sports performance [18, 55, 57, 61]. Having been identified as offering a valid theoretical explanation of team sport performance and an improved approach to performance analysis [62], ecological dynamics may prove a suitable lens through which rugby league performance can be better understood. The seminal work of Gibson [60] describes the theoretical basis of ecological dynamics. When team sports are viewed through an ecological dynamics lens, match-play is conceptualised as a *complex adaptive system* [63], where multiple individuals are interacting within an environment. A key concept of this is that information is detected (within an optical array) and attuned to by individuals from the environment, allowing for the perception of affordances (i.e. opportunities for action [55]) and realisation of affordances (e.g. an action or decision) [64]. Thus, there is constant regulation between information, perception and action. Importantly, these dynamical systems are subject to constraints, including task, individual and environment classes (Fig. 2). Where these constraints interact may therefore be thought of as a dynamic and ever-changing adjustment, regulated by exposure to these interactions [65]. On this understanding, the perception of affordances acts as a governor, restricting the number of action opportunities, at that point in time [60]. As opportunities are taken (causing an action), further information becomes available to attune to, allowing for continuous affordance perception (opportunities for action) that may influence subsequent affordance realisation (actions) (Fig. 3). Notably, embedded in this theory is the notion that performers can be trained to become perceptually attuned in specific environments, enabling effective action opportunities (i.e. a learning effect) [66].

**Fig. 4:**
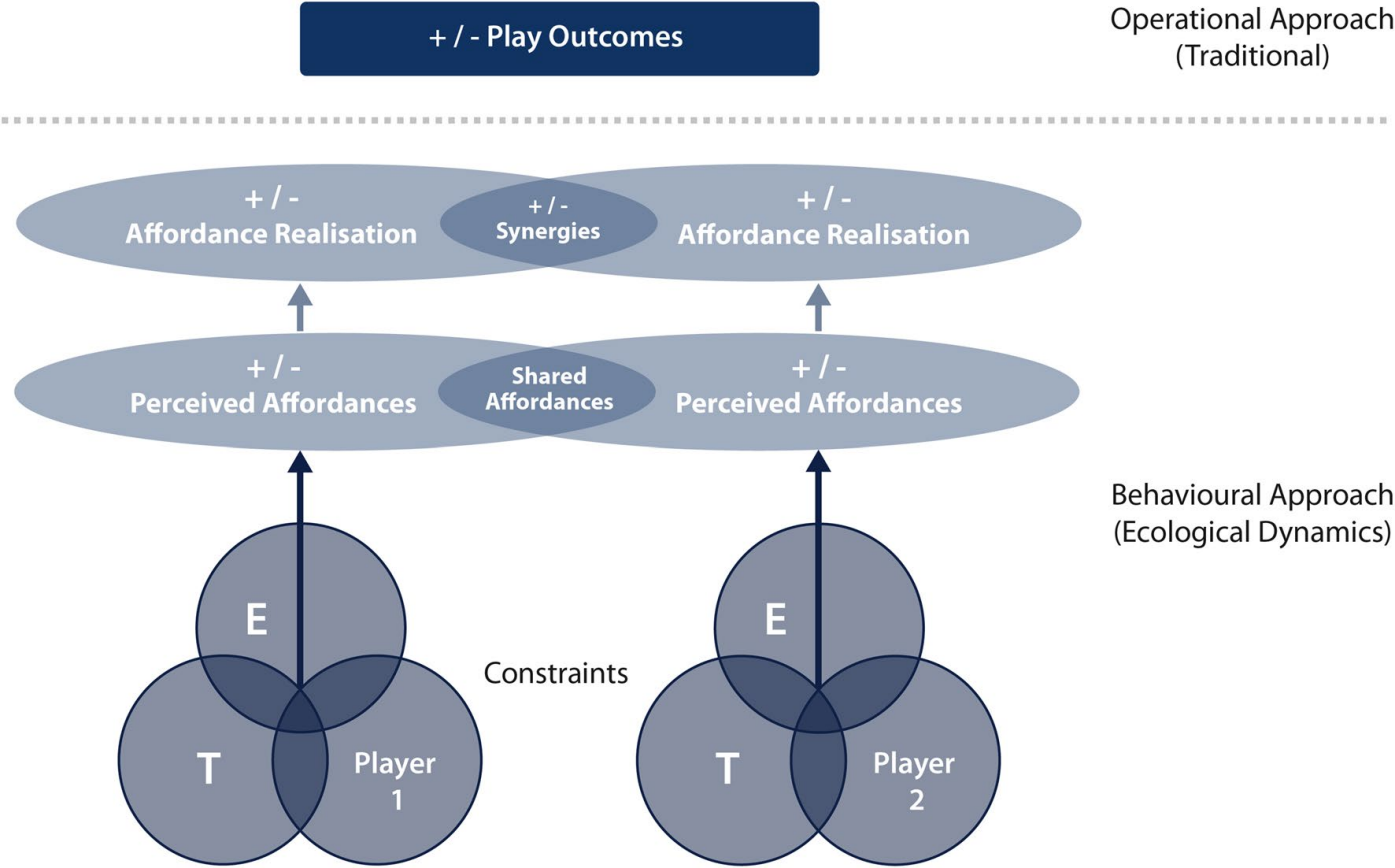
Theoretical basis of shared player behavioural regulation within an ecological dynamics framework. (E) The environment; (T) the task being performed; and the individual player

Given a team sport player’s actions, action capabilities and even their intentions alone are readily perceived by other players, and in turn acted upon (continuously); players are considered to be connected informationally (and on occasion mechanically in rugby league). This connectedness results in observable and measurable group dynamics (e.g. dynamic systems theory), where action opportunities are shared, permitting coordinated team behaviours to emerge (Fig. 4). Indeed, collective (or shared) affordances can be perceived by a group of individuals (teammates), who may be attuned to perceive them [58]. Equally, should one member of the team not perceive these affordances as other team members do, the action opportunity is not ‘shared’ and this team member may find themselves ‘out of position’; as a result, the coordinated team behaviour may never emerge (if earlier in the play) or it may break down (if later in the play). These shared affordances may be considered as either those that an individual may present to others within the environment (i.e. affordances *for* teammates), or the affordances other individual actions provide the perceiver (i.e. affordances *of* teammates) [67]. Cohesive and coordinative team behaviour is therefore dependent on the ability of individuals to collectively attune to these shared affordances. Importantly for practitioners, the ability to attune to these shared affordances (both *for* and *of* teammates) can be improved through training and match-play, as individuals manage and align their behaviours (actions) within the constraints of the environment, task and other individuals [58]. Indeed, this alteration in perceptual attunement is supported by system degeneracy, whereby individuals may effectively govern and manipulate their behaviours in independent ways to garner a similar (or desired) outcome. For example, an individual may learn to more appropriately adjust their movement patterning (e.g. accelerate), based on their enhanced ability to distinguish which detected information to attune to in specific performance situations that may be beneficial to a performance outcome (e.g. line break) [68]. The perception and realisation of such shared affordances allows for synergistic and cohesive team behaviours.

Within a dynamical systems theoretical framework, the emergence of these coordinated behaviours in team sports (such as rugby league) is founded on the interactive *synergies* between individual players [58]. Here, synergies may be defined as a group of relatively independent degrees of freedom that behave as a single functional group [69] (i.e. the result/outcome of individuals' collective actions, established on shared affordances) [58]. In rugby league terms, players would form a synergy when they (as the degrees of freedom) combine to perform a function together. Importantly, synergies possess certain properties that when measured describe the emergence, persistence and decay of self-organised, coordinated team behaviours (i.e. actions at a macroscopic level) [55]. Collectively, this offers insights into the functional utility of player behaviours (the *why?* and *how?*), which builds upon the *descriptions* of performance obtained through more traditional notational analysis (separate analysis of individual players) [51].

Conceptualised through the ecological dynamics framework described in Fig. 4, team match-play is positioned as a collection of players continuously self-organising their behaviours (motor behaviours) in response to the shared action opportunities perceived in the performance environment. Further to this, and of benefit in team sports, ecological dynamics can be applied at varying timescales. For example, this model can be applied to understand how three players combine to orchestrate a line break during play.

### A Novel Model of Rugby League Performance

**Fig. 5:**
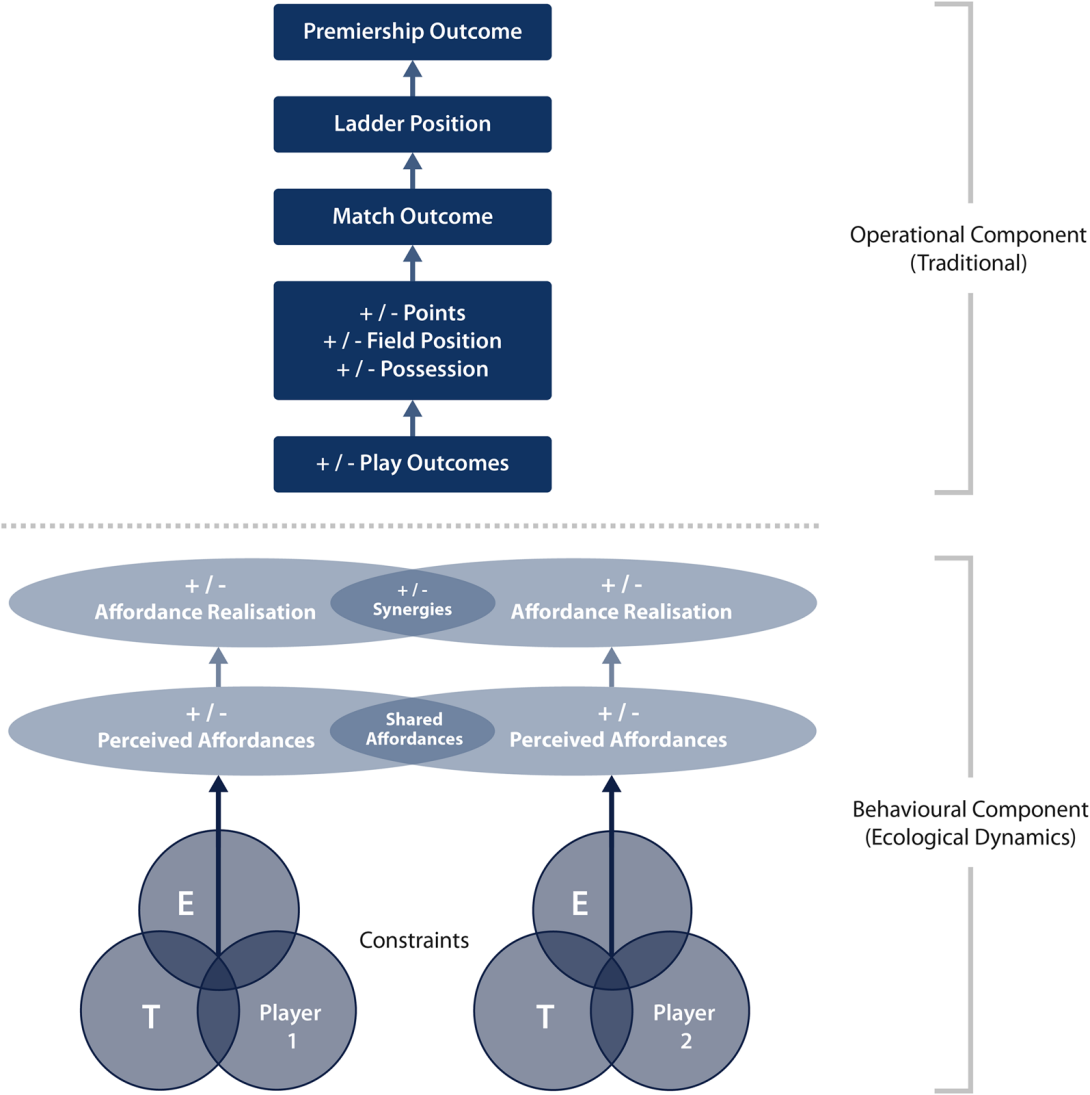
Proposed novel model of Rugby League performance; integrating hierarchical invasion sports and ecological dynamics theory. (E) The environment; (T) the task being performed; and the individual player

To define and evaluate rugby league performance it appears necessary to understand the operational outcomes (hierarchical model) alongside the underpinning team and individual behaviours (ecological dynamics). Through conceptualising rugby league performance using these outputs and inputs, we may explore current evidence to establish the KPIs, as they relate operationally, and the key inputs (i.e. the individual, the environment and the task). The outcome of such an exploration would benefit the improvement in player (and team) preparation and development frameworks, providing a unique method to establish systems without defined disciplines (eliminating silos). Indeed, there is promise that exploring rugby league performance through this lens can assist practitioners (i.e. tactical coaches, performance staff) to evaluate and define aspects of performance traditionally left for skill acquisition and motor control experts. As such, we propose a rugby league performance model conceptualised through the ecological dynamics framework (Fig. 5). This figure illustrates a theoretical (behavioural) approach that may underpin our understanding of match-play success, both within matches and over a prolonged period. Importantly, this conceptualisation may be extended more widely to other team sports due to its generalised holistic approach.

### Applications of the Novel Rugby League Performance Model

As noted, the proposed model may be implemented within micro- (specific match-play tasks) and macro-structures (preparation and development plans). Through examples, this section aims to demonstrate its utility across timescales.

#### Performance Analysis to Understand Play Outcomes

A *dummy half run* is an example of an individual action that is recorded in traditional match analyses. For greater insight, performance staff may use the parameters presented in Fig. 5 to appraise the appropriateness of such actions. Accordingly, a dummy half run may occur when relevant information is detected and attuned to, and affordances are perceived and realised (resulting in the action). In this specific circumstance, the dummy half will detect and attune to pertinent information from the environment; for example, there may be an opposition marker left on the ground during the tackle. Shaped and constrained by the interaction between the task (e.g. promote the ball downfield), the environment (e.g. field position, weather, the fallen opposition player) and the individual (e.g. their capabilities), the dummy half perceives an affordance (opportunity for action). As such, a realisation of this affordance (an action) occurs, as the dummy half accelerates towards the space behind the ruck (play the ball). We may also view this specific example at a team level, to understand how shared affordances and team synergies are developed. Indeed, while a player electing to run from dummy-half may be identified as an individual action, in reality it forms part of the team’s collective motion, due to the fact that other players recognise the action and alter their behaviour accordingly. Imagine another attacking player (e.g. the fullback) is attuned to the same information, perceiving the affordance of the dummy half run (again, shaped by their own interaction with the task, environment and individual). This shared recognition of the opportunity that exists can be conceptualised as a *shared affordance*. In this example, just prior to the dummy half running, the fullback accelerates (affordance realisation), creating a shared action (*team synergy*). Ultimately, the process of both players detecting and attuning to the same information, perceiving an affordance in a similar manner, creates shared affordances that may lead to appropriate affordance realisation and synergistic actions which complement each other. Yet this sequence can break down at any number of moments, as highlighted earlier, emphasising the importance of the perception and realisation of affordances in task outcome.

### Reconceptualising the Practitioner's Role: Implications for Preparation and Development

On the understanding that the primary goal of rugby league player preparation and development programmes is to improve performance, the proposed rugby league performance model brings into question the appropriateness of current practice, where themes of work (technical, tactical, physical, mental) are designed and implemented. Importantly, recent evidence in rugby league suggests that there exists a mismatch between qualities (physical) deemed important by coaches and specific coaching practice to stimulate relevant adaptations [70]. While this may not be unique to rugby league, it demonstrates that there may be a need to better align our practices and training rationale/methods. Such a change in philosophy might require the elimination of established silos in which our preparation and development models traditionally work, allowing for greater horizontal and vertical integration and transfer of information [19]. Through this framework, all coaches (tactical, technical, performance, etc.) may view themselves as a *designer*. As a designer, coaches create environments for player development, harnessing the continuous, nonlinear and integrated interactions that emerge between the environment, task and individual [18, 71]. This approach may also shift away from the externally driven (re)organisation of constraints applied to athlete–environment systems (traditional coach/teacher), instead providing greater opportunity for self-regulation and exploration from internally driven sources [18, 61]. Indeed, through deliberate training *design*, players can utilise temporally structured, functional actions (perception–action coupling) to self-regulate through unexplored landscapes, composed of emergent problems (termed ‘wayfinding’) [61]. This conceptualisation of coach roles, players and the training experience, is key if players are to express their individual capabilities successfully in match-play. Indeed, if this type of development is not afforded, we will no longer see examples of performers/athletes approaching situations in innovative/different ways. Indeed, performances previously deemed ‘impossible’ or ‘radical’ (e.g. Roger Bannister conquering the 4-min mile [72] and Dick Fosbury revolutionising the high jump technique) will unlikely be sought to be challenged or explored.

#### Learning Environment Designers on the Field

Through an exploration and revision of the training plan, coaches can design information rich practice tasks that encourage the player-environment interaction. When viewed through this *designer* lens coaches seek to support the growth of players specific to the affordances perceived within their environment [73]. Given player preparation and development programs are multidisciplinary, it is fundamental to this approach that sub-disciplinary support roles (e.g. strength and conditioning coach, sports scientists, performance analysts, specialist coaches) are to be reconceptualised as learning environment designers, responsible for the co-design of these landscapes [11]. By doing so, high-performance support staff may enhance the relevant selection and integration of environmental constraints to ensure the training design promotes affordance realisation, appropriately matching with the action capabilities of individual players [74]. For example, when undertaking tactical-based training, practitioners can utilise their expertise to allow for an athlete–environment interaction that considers the tactical, physiological and psychological demands of competition [11]. Such co-design may include integrated information from: (1) sports scientists to identify specific movement and collision demands at certain intensities and periods of match-play; (2) strength and conditioning coaches to provide detail on replicating and stimulating the associated physiological response of specific situations; (3) specialist attacking coaches to guide the search of players to manipulate the defence into a system of play they can exploit (e.g. repeatedly forcing specific players into tackling at certain positions of the field to gain an over-lap). Indeed, utilising nonlinear pedagogical approaches (such as this representative learning design), coaches can support the growth and development of individuals in their environment. Furthermore, including and engaging individual players in the development of their practice tasks (e.g. representative co-design) can promote individual responsibility for these tasks, as well as expanding their understanding of the performance environment [74]. Through the provision of co-designed environments and tasks, players may enhance their ability to detect and attune to information, alongside their perception and realisation of (shared) affordances under the constraints specific to match-play conditions.

#### Learning Environment Designers in the Gym

Notably, this approach can also be applied to other areas of the player preparation and development program. For example, in strength and power development, practitioners may typically apply a constraint-led approach, whereby they regularly constrain the affordances to reduce available actions (to varying degrees). While there may be elements of programming where this is deemed necessary (e.g. the development of fundamental lower-body strength qualities through a back squat), we may also consider the application of physical qualities (movement patterns) that have a greater transference to environment specific actions and promote *wayfinding* [61]. Indeed, we can perturb movement through various means that alter the degrees of freedom afforded to the athlete. Through even a small shift in some of our training design practices we may allow athletes to find alternative ways (affordances) to solve movement problems (affordance realisation), through the self-exploration of certain tasks. An example of this may be the implementation of more broad physical literacy in youth athletes [75, 76]. Physical literacy has been described as ‘a multifaceted conceptualisation of the skills required to fully realise potentials through embodied experience’ [77], supported by an individual’s movement capacities (including balance, co-ordination, flexibility, agility, control, precision, strength, power, endurance and speed) [78]. Within the framework of ecological dynamics we may construct and structure enriched environments to promote an athlete–environment interaction that supports the acquisition of functional movement skills [76]. Specific to a development programme, practitioners may include elements of physical literacy alongside alternative methodologies and theoretical perspectives (e.g. dynamic systems) to develop, periodise and progress physical development. Here, coaches could integrate a constraint-led approach, manipulating the constraints applied to individuals, offering affordances that allow these youth athletes to navigate their landscape within different tasks [75]. Over time (i.e. a periodised programme), practitioners might choose to progressively add complexity (e.g. task complexity, load and intensity), yet must be cognisant of the constraints applied and the subsequent restrictions of affordances. Through these training designs coaches may not only enhance athlete dexterity but improve the ability to search, detect and attune to information towards certain tasks, environments and their own individual action capabilities.

## General Conclusions

This paper aimed to provide coaches and performance staff with an evidence-based framework of rugby league performance, evaluating a traditional approach and conceptualising a novel, modern approach. Based on a traditional hierarchical model of performance, a breadth of performance focussed rugby league literature was reviewed to examine what insights have been gained from notational methods explored in a linear fashion. While a selection of KPIs exist, they are intuitive, unsurprising and highlight clear gaps in our understanding of how players’ capabilities combine as a team to deliver a performance. Moreover, there is clear evidence to suggest rugby league competitions are not static, but rather constantly evolving and therefore so are rugby league performance indicators. Part 1 of this review demonstrated a need to identify the mechanisms that govern the expression of players’ individual capabilities during play, if a meaningful understanding of rugby league game play is desired.

Consequently, we conceptualised rugby league performance through the lens of ecological dynamics, describing the foundational importance of the deeply integrated interactions between dynamical systems. Through this lens, we may offer an explanation of the underpinning *team behaviour* that feeds up into the hierarchical approach. We suggest it is the exposure of the player to an ecologically challenging environment, where they are afforded opportunities around their task and the environmental situational/game conditions, which will then allow learning within the training/playing environment. As a result, it is the development of *individual capabilities*, through directed training systems that will lead to the application of effective affordance realisation and appropriate individual and team actions (team synergy). Notably, the proposed performance model may be used across other team sports providing practitioners with greater insights into understanding the *how* and *why* of individual and team behaviour (and success). We aimed to offer contextual examples of how conceptualising rugby league performance in this light could support the advancement of preparation and development frameworks. While these examples are not exhaustive, we hope they provide practitioners with the contextual information to allow for the appropriate implementation of the proposed model in rugby league. In addition, we implore other researchers and practitioners to continue to challenge the operational approach to performance in rugby league, removing current ‘silo’ coaching frameworks and instead developing a shared and athlete-focussed approach [11, 19]. While further evidence is required to develop these methods, we believe this review provides a unique outline on which rugby league performance may be described and player preparation and development frameworks conceptualised.

## Data Availability

Not applicable.

## Abbreviations

KPI: Key performance indicator
NRL: National Rugby League
NYC: National Youth Competition
SL: Super League
